# Volatile organic compounds in ventilated critical care patients: a systematic evaluation of cofactors

**DOI:** 10.1186/s12890-017-0460-0

**Published:** 2017-08-22

**Authors:** Tobias Hüppe, Dominik Lorenz, Mario Wachowiak, Felix Maurer, Andreas Meiser, Heinrich Groesdonk, Tobias Fink, Daniel I. Sessler, Sascha Kreuer

**Affiliations:** 1 Department of Anaesthesiology, Intensive Care and Pain Therapy, Centre of Breath Research, Saarland University Medical Centre, Kirrberger Strasse 100, 66421 Homburg (Saar), Germany; 2 Department of Anaesthesiology and Intensive Care, Klinikum Lünen St.-Marien-Hospital, Lünen, Germany; 3 Department of Outcomes Research, Anesthesiology Institute, ASCleveland Clinic, Cleveland, OH USA

**Keywords:** Volatile organic compound, Anaesthesia, Critical care, Breath analysis, Mechanical ventilation

## Abstract

**Background:**

Expired gas (exhalome) analysis of ventilated critical ill patients can be used for drug monitoring and biomarker diagnostics. However, it remains unclear to what extent volatile organic compounds are present in gases from intensive care ventilators, gas cylinders, central hospital gas supplies, and ambient air. We therefore systematically evaluated background volatiles in inspired gas and their influence on the exhalome.

**Methods:**

We used multi-capillary column ion-mobility spectrometry (MCC-IMS) breath analysis in five mechanically ventilated critical care patients, each over a period of 12 h. We also evaluated volatile organic compounds in inspired gas provided by intensive care ventilators, in compressed air and oxygen from the central gas supply and cylinders, and in the ambient air of an intensive care unit. Volatiles detectable in both inspired and exhaled gas with *patient-to-inspired gas ratios* < 5 were defined as *contaminating* compounds.

**Results:**

A total of 76 unique MCC-IMS signals were detected, with 39 being identified volatile compounds: 73 signals were from the exhalome, 12 were identified in inspired gas from critical care ventilators, and 34 were from ambient air. Five volatile compounds were identified from the central gas supply, four from compressed air, and 17 from compressed oxygen. We observed seven *contaminating* volatiles with *patient-to-inspired gas ratios* < 5, thus representing exogenous signals of sufficient magnitude that might potentially be mistaken for exhaled biomarkers.

**Conclusions:**

Volatile organic compounds can be present in gas from central hospital supplies, compressed gas tanks, and ventilators. Accurate assessment of the exhalome in critical care patients thus requires frequent profiling of inspired gases and appropriate normalisation of the expired signals.

## Background

Multi-capillary column ion-mobility spectrometry (MCC-IMS) can be used for real-time clinical breath analysis [1]. Volatile organic compounds in expired gases (exhalome) are linked to physiological processes and various diseases [2, 3]. It may also be possible to estimate plasma drug concentrations from the exhalome [4]. For example, there is great potential to be able to diagnose lung cancer, chronic obstructive pulmonary disease (COPD), lung infections, and renal failure which all need to be confirmed in clinical trials [3]**.** Exhalome analysis may also facilitate early detection of inflammation and sepsis — although this application has so far only been evaluated in rats [5].

Accurate assessment of volatile organic compounds in expired gas requires either that none be present in inspired gas or that inspired concentrations are measured and subtracted from the raw expired signal. Potential sources of exogenous volatile organic compounds include gas supplies (central hospital, compressed cylinders, ambient air) and ventilators.

Our concern was prompted by a previous study in which there was substantial contamination of inspired gas [6], including inhaled volatile compounds that were subsequently exhaled unchanged and might thus have been mistaken for biomarkers. We therefore evaluated volatile organic compounds in ambient air in a critical care unit, in gases from the central hospital supply as well as in compressed gas cylinders and from critical care ventilators.

## Methods

### Patients

With approval by the responsible ethics committee (Ärztekammer Saarland, Saarbrücken, Germany Ref-No. 232/14), five sedated and mechanically ventilated adults were each evaluated for 12 h. Legal guardians or the patients themselves subsequently agreed in written form to participate in this study.

Patients were ventilated with an intensive care respirator (EVITA 4, Dräger, Lübeck, Germany) with ventilation parameters and oxygen concentrations adjusted to maintain physiological partial pressures of carbon dioxide and oxygen. They were ventilated with either pressure-supported or pressure-controlled modes. A MCC-IMS aspiration tube was connected to the endotracheal tube distal to a heat-and-moisture exchanging filter (Humid-Vent Filter Compact S, Teleflex Medical, Athlone, Ireland) by a polytetrafluoroethylene tube (Bohlender, Grünsfeld, Germany). Samples were aspirated from the breathing circuit at 30-min intervals.

### Ventilators and gas sources

We evaluated three different isolated EVITA 4 ventilators (Dräger, Lübeck, Germany), each provided by gas (oxygen and compressed air) from central hospital supplies. Each ventilated an artificial lung at a minute volume of eight litres per minute for 12 h with an oxygen fraction of 21%. Gas was sampled at 30-min intervals from tubing connected to a heat-and-moisture exchanging filter which was connected to the outlet port of the ventilator.

We also evaluated oxygen and compressed air provided by our hospital’s central gas supply. Gases were passed through a pressure regulator into polytetrafluoroethylene tubing and then into a five-litre glass bottle at a rate of 1.0–1.2 l per minute. To avoid contamination of ambient air, the glass container was sealed towards the outside and flushed with oxygen or compressed air for 60 min before each set of measurements. Oxygen and air from the hospital central supply system were each evaluated from three different wall distribution stations. We also evaluated compressed oxygen from three different cylinders using the method mentioned above. In each case, samples of gas from the glass bottles were taken at 30-min intervals for 12 h.

And finally, we evaluated ambient air in an ICU on three distinct days, each for 24 h with a sampling interval of 30 min.

### Analysis of volatile organic compounds

Volatile organic compounds in all gas samples were evaluated as described previously using a BreathDiscovery MCC-IMS (B&S Analytik, Dortmund, Germany) [6, 7]. Briefly, 10-ml gas samples were analysed in multicapillary columns which evaluated compound retention times (RT) and combined with ion-mobility spectrometry also evaluated drift times. IMS-Peaks with an intensity of more than 5 mV in at least three consecutive measurements were included. Volatile organic compounds were thus characterised by their retention times and drift times which identify specific compounds, and peak intensity which is a function of concentration.

Specific volatile compounds were identified using the software Visual Now 3.6 (B&S Analytik, Dortmund, Germany) by automated alignment software (MIMA, version 1.1.2) with an existing database (BS-MCC/IMS-analytes database, version 1209, B&S Analytik, Dortmund, Germany) [8]. Peak area overlapping of at least 10% with preexisting reference substance in chromatogram defined alignment. If overlapping areas of two eligible compounds differed less than 10% in extent, the alternative compound was designated as well. Unknown volatiles were designated only by unique peak numbers. We performed calibrations for the volatiles acetone, cyclohexanone, dimethyl disulphide, 3-hydroxy-2-butanone, 2-methylfuran, 2-methylpentane, and 3-pentanone using exponential dilution technique with a 5.9 l glass bottle as described previously [9].

Intensities of different volatile organic compounds were expressed as means (± 95% confidence interval) of the relevant sampling periods. As in a previous study, we classified volatiles as *expired* (detected only in expired gas or having a *patient-to-inspired gas ratio* > 1.5), *unaffected* (having a *patient-to-inspired gas ratio* between 0.5–1.5), and *resorbed* (having a *patient-to-inspired gas ratio* < 0.5) [6]. Gases with *patient-to-inspired gas ratios* < 5 were considered clinically important because they might be mistaken for de novo expired compounds.

## Results

**Fig. 1:**
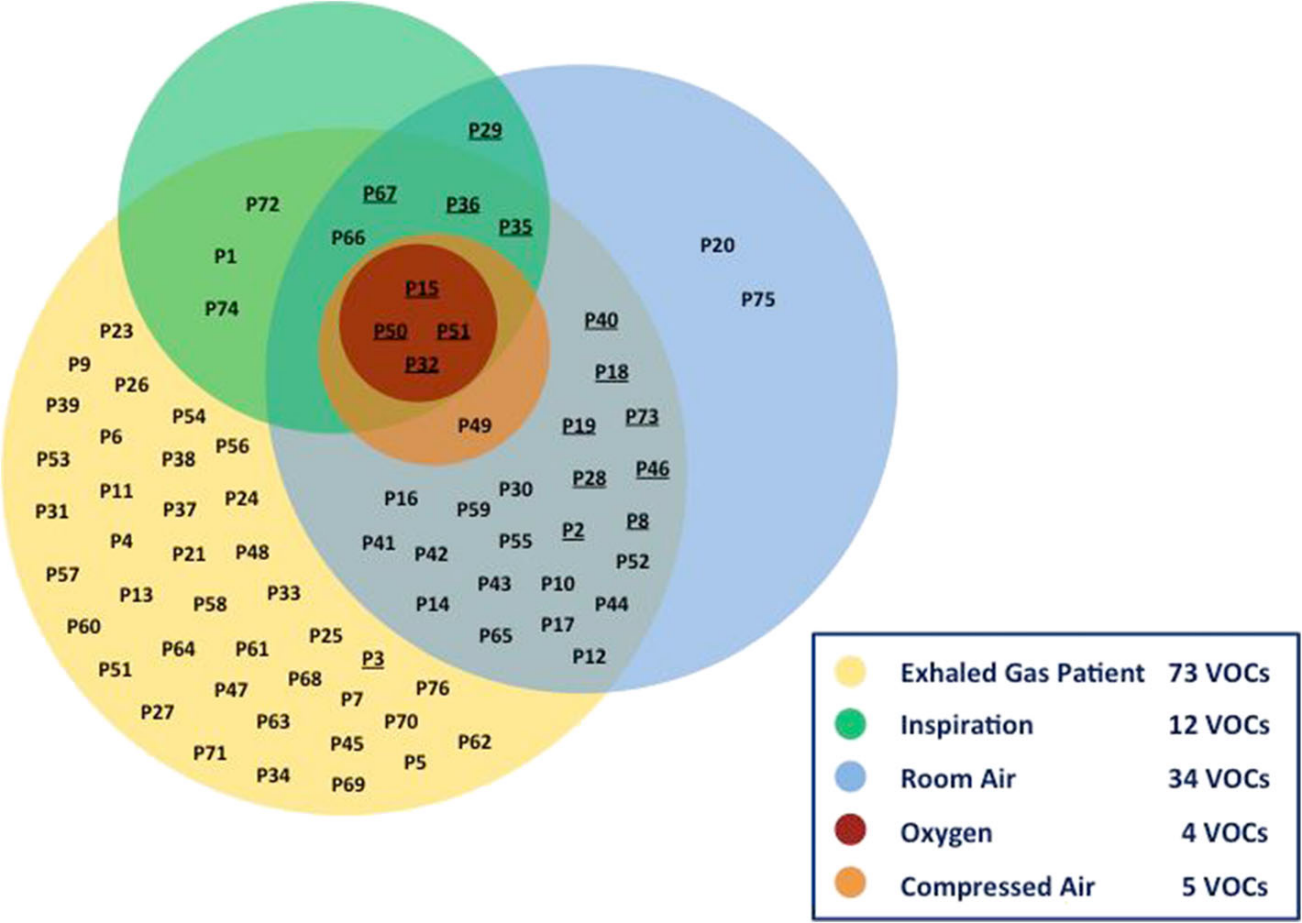
Occurrence and intersecting sets of all detected signals. Underlined peaks = additional occurrence in oxygen from cylinder (*n* = 17)

A total of 76 different signals were detected by MCC-IMS of which 48 were identified. Volatile compounds and their peak numbers in the IMS chromatogram, CAS number, class, and chemical identification (when known) are shown in Table 1. Seven peaks aligned with each two eligible reference substances. Table 2 shows intensities of all detected volatiles according to different sampling points. Concentrations in ppb of selected compounds are summarized in Table 3. Figure 1 displays the occurrence and intersecting sets of all signals.

**Table 1:** Peak number in the IMS chromatogram, volatile organic compound, CAS number (Chemical Abstracts Service), class and occurrence of chemical substance; (*) = alternative volatile organic compound; P49 – P76 are “unknown” signals and are not displayed

	Chemical Substance			
IMS-Peak	Volatile Organic Compound	CAS number	Class of chemical substance	Occurrence
P1	Acetone *monomer*	67–64-1	Ketones	Synthesis with a raw material, solvents, adhesives
P2	Acetone *dimer*	67–64-1	Ketones	Synthesis with a raw material, solvents, adhesives
P3	Benzofuran	271–89-6	Aromatics	Tabacco smoke, synthesis chemicals
P4	Butanal *monomer* (1-Butanol*)	123–72-8	Aldehydes	Artificial resin, plasticizer
P5	Butanal *dimer*	123–72-8	Aldehydes	Artificial resin, plasticizer
P6	1,2-Butandiol	584–03-2	Alcohol	Solvents, epoxy resins
P7	2,3-Butandiol	513–85-9	Alcohols	Solvents, plasticizers, epoxy resins, toiletries
P8	2-Butanone	78–93-3	Ketones	Solvents, plastics, sterilization of medical products
P9	(+)Camphene	79–92-5	Terpenes	Ethereal oils
P10	Cyclohexanol *monomer* (3-Heptanon*)	108–93-0	Alcohols	Solvents
P11	Cyclohexanol *dimer*	108–93-0	Alcohols	Solvents
P12	Cyclohexanone *monomer*	108–94-1	Ketones	Solvents
P13	Cyclohexanone *dimer*	108–94-1	Ketones	Solvents
P14	p-Cymol	99–87-6	Terpenes	plants
P15	Dimethyl disulphide *monomer*	624–92-0	Disulphide	Flavouring
P16	Dimethyl disulphide *dimer*	624–92-0	Disulphide	Flavouring
P17	2,5-Dimethylpyrazin	123–32-0	Azine	Food, flavouring
P18	Ethanol	64–17-5	Alcohols	Fermentation, disinfectant, solvents
P19	Ethylbenzene	100–41-4	Aromatics	Solvents, plastics, lacquers
P20	2-Ethyl-1-hexanol	104–76-7	Alcohols	Solvents, intermediates
P21	Heptanal	111–71-7	Aldehydes	Intermediates, odor agents
P22	2-Heptanone	110–43-0	Ketones	High boiling solvents, coating material
P23	3-Heptanone (4-Heptanone*)	106–35-4	Ketones	Solvents
P24	Hexanal	66–25-1	Aldehydes	Lipid peroxidation of unsaturated fatty acids
P25	1-Hexanol	111–27-3	Alcohols	Solvents, plasticizer
P26	2-Hexanol	626–93-7	Alcohols	Solvents
P27	2-Hexanon (Hexanal*)	591–78-6	Ketones	Solvents
P28	3-Hydroxy-2-Butanone	513–86-0	Ketones	Bacteria, tobacco smoke
P29	Isoprene *monomer*	78–79-5	Terpenes	rubber
P30	Isoprene *dimer*	78–79-5	Terpenes	rubber
P31	Menthone	10,458–14-7	Ketones	Ethereal oils
P32	Methanol	67–56-1	Alcohols	Solvents, synthesis with a raw material
P33	3-Methylbutanal	590–86-3	Aldehydes	Drug substances, vitamins, solvents, plasticizers
P34	2-Methylbutylacetat (Hexanal*)	624–41-9	Acetic Esters	Solvents, flavouring
P35	2-Methylfuran	534–22-5	Furans	Tobacco smoke
P36	2-Methylpentane	107–83-5	Hexane	Solvents, cleaning agents
P37	n-Nonane	111–84-2	Alkanes	Fuels, Entrainer, detergent substances
P38	2,2,4,6,6-Pentamethylheptane	236–757-0	Alkanes	Solvents, cleaning agents
P39	1-Pentanol (Cyclohexanol*)	71–41-0	Alcohols	Solvents, cleaning agents, disinfectant
P40	2-Pentanone	107–87-9	Ketones	Solvents
P41	3-Pentanone *monomer*	96–22-0	Ketones	Solvents
P42	3-Pentanone *dimer*	96–22-0	Ketones	Solvents
P43	Phenylacetylene *monomer* (Dimethyl disulphide*)	536–74-3	Alkynes	Plastics
P44	Phenylacetylene *dimer*	536–74-3	Alkynes	Plastics
P45	1-Propanol	71–23-8	Alcohols	Solvents, disinfectant, cleaning agents
P46	2-Propanol *monomer*	67–63-0	Alcohols	Solvents, cleaning agents, disinfectant
P47	2-Propanol *dimer*	67–63-0	Alcohols	Solvents, cleaning agents, disinfectant
P48	Propofol	2078–54-8	Phenol	Anaesthetic

**Table 2:** Peak intensities of all detected volatile organic compounds detected in gas from designated sources

IMS-Peak	Volatile Organic Compound	Patient		Inspiration		O_2_		Compressed Air		O_2_ Cylinder		Room Air	
P1 *	Acetone monomer	71	(60.1–81.9)	6	(4.8–7.3)								
P2 *	Acetone dimer	137	(91–183)							5	(3.4–6.6)	12.1	(10.6–13.6)
P3 *	Benzofuran	4.9	(3.2–6.5)							2.7	(2–3.4)		
P4 *	Butanal monomer	7.6	(6.2–9)										
P5 *	Butanal dimer	4.6	(3.8–5.3)										
P6 *	1.2-Butandiol	5	(4.2–5.8)										
P7 *	2.3-Butandiol	6.9	(5.9–7.8)										
P8 *	2-Butanone	15.2	(10.5–19.9)							25.8	(14–37.6)	3.2	(2.8–3.6)
P9 *	(+)Camphene	8.8	(7.2–10.4)										
P10 *	Cyclohexanol monomer	17.7	(12.3–23.1)									10.3	(9.2–11.4)
P11 *	Cyclohexanol dimer	8.9	(7.8–10)										
P12 *	Cyclohexanone monomer	175	(160–190)									9.7	(9.3–10.1)
P13 *	Cyclohexanone dimer	4	(3.4–4.6)										
P14 *	p-Cymol	4	(3.1–4.8)									4	(3.8–4.3)
P15 *	Dimethyl disulphide monomer	88	(68.9–107)	10	(8.7–11.3)	34.2	(32.8–35.6)	8.8	(7.5–10)	18.3	(16.2–20.4)	44.4	(42.3–46.5)
P16 *	Dimethyl disulphide dimer	7.6	(6.3–8.9)									12.7	(12–13.4)
P17 *	2.5-Dimethylpyrazin	98.3	(58.9–138)									7.6	(7–8.2)
P18 *	Ethanol	5.5	(2.9–8.2)							9.1	(6.6–11.7)	26.7	26.7
P19 *	Ethylbenzene	35.8	(22–49.6)							6.2	(3.8–8.6)	12.2	(11.2–13.2)
P20	2-Ethyl-1-hexanol											4.1	(3.9–4.4)
P21 *	Heptanal	9.9	(9–10.9)										
P22 *	2-Heptanone	13.5	(11.9–15.1)										
P23 *	3-Heptanone	20.4	(18.2–22.6)										
P24 *	Hexanal	7.4	(6.8–8)										
P25 *	1-Hexanol	10.5	(8.8–12.2)										
P26 *	2-Hexanol	16.2	(13.4–19)										
P27 *	2-Hexanone	7.1	(6.3–7.9)										
P28 *	3-Hydroxy-2-Butanone	66.8	(43.7–89.9)							1.9	(1.5–2.2)	86.6	(76.6–96.6)
P29 ^§^	Isoprene monomer			8.1	(7.7–8.5)					54.2	(31.2–77.2)	10.6	(8.8–12.4)
P30 *	Isoprene dimer	4.5	(3.7–5.3)									3.6	(3.3–3.8)
P31 *	Menthone	14.5	(12.9–16.1)										
P32 *	Methanol	107	(102–112)	45.9	(36.8–55)	98.2	(96.7–99.7)	87.4	(85.3–89.5)	101	(97.2–105)	98	(97–99)
P33 *	3-Methylbutanal	11.2	(9.3–13.1)										
P34 *	2-Methylbutylacetat	7.1	(6.3–7.8)										
P35 *	2-Methylfuran	26.2	(15.3–37.1)	3.9	(3.5–4.4)					3.4	(2.3–4.5)	13.8	(11.9–15.7)
P36 *	2-Methylpentane	15.3	(10.4–20.2)	6	(5.2–6.8)					51.3	(29.2–73.4)	27.8	(24.1–31.5)
P37 *	n-Nonane	83.4	(66.5–100)										
P38 *	2.2.4.6.6-Pentamethylheptan	7.7	(6.3–9)										
P39 *	1-Pentanol	17.9	(12.3–23.5)										
P40 *	2-Pentanone	130	(86.8–173)							5.7	(4–7.5)	3.8	(3.5–4.1)
P41 *	3-Pentanone monomer	230	(167–294)									35.5	(31.7–39.3)
P42 *	3-Pentanone dimer	21.1	(20–22.2)									5.6	(5.5–5.7)
P43 *	Phenylacetylene monomer	96.9	(79.2–115)									5	(4.7–5.3)
P44 *	Phenylacetylene dimer	19.4	(15.3–23.5)									6.1	(5.8–6.5)
P45 *	1-Propanol	5.8	(4.8–6.8)										
P46 *	2-Propanol monomer	22	(17.2–26.8)							11.4	(6.8–16)	44.9	(38.8–51)
P47 *	2-Propanol dimer	4.7	(4–5.5)										
P48 *	Propofol	23.2	(19.6–26.8)										
P49 *		36.2	(33.1–39.3)					4.5	(4.1–5)			24.3	(24–24.6)
P50 *		43.6	(34.6–52.6)	17.5	(15.4–19.7)	6.1	(4.6–7.6)	5.1	(3.8–6.5)	2.8	(1.7–3.8)	2.7	(2.1–3.2)
P51 *		20.9	(14.5–27.3)	12.2	(10.2–14.2)	4.9	(3.7–6)	4.3	(3.2–5.3)	0.9	(0.2–1.6)	2.1	(1.7–2.4)
P52 *		15.8	(12.3–19.4)									9.1	(8.3–10)
P53 *		13.8	(12.9–14.7)										
P54 *		4.6	(3.9–5.3)										
P55 *		43.5	(37.8–49.2)									3.1	(2.3–4)
P56 *		7.1	(5.7–8.5)										
P57 *		9.9	(6.9–13)										
P58 *		2.4	(2–2.7)										
P59 *		3.5	(1.2–5.7)									3.7	(3.5–4)
P60 *		4.7	(4.1–5.4)										
P61 *		5.3	(3.6–7)										
P62 *		2.8	(2.3–3.3)										
P63 *		3.7	(3–4.3)										
P64 *		5.1	(3.9–6.4)										
P65 *		5.9	(3.6–8.3)									8.4	(7.6–9.2)
P66 *		6.2	(4–8.3)	3.2	(2.8–3.7)							4.8	(4.2–5.3)
P67 ^#^		3.7	(1.1–6.2)	3.5	(3.1–4)					4.9	(3.5–6.3)	5.8	(5.1–6.5)
P68 *		1.8	(1.3–2.2)										
P69 *		4	(2.6–5.3)										
P70 *		8	(3.4–12.6)										
P71 *		2.1	(1.5–2.6)										
P72 *		15.6	(13.9–17.3)	3.1	(2.5–3.6)								
P73 *		4.6	(2.3–6.8)							3.7	(2.6–4.8)	4.5	(4.2–4.8)
P74 ^#^		2.4	(2–2.8)	3.1	(2.6–3.6)								
P75												8.7	(8.1–9.2)
P76 *		8.6	(6.8–10.5)										

Results are shown as means (95% confidence interval) in millivolt [mV]; Peak 49–76 represents “unknown” compounds; * = *expired* compounds, # = *unaffected* compounds, § = *resorbed* compounds

**Table 3:** Peak intensities and corresponding concentration [ppb] of selected volatile organic compounds

Volatile Organic Compound	Patient		Inspiration		O_2_		Compressed Air		O_2_ Cylinder		Room Air	
	mV	ppb	mV	ppb	mV	ppb	mV	ppb	mV	ppb	mV	ppb
Acetone	345 (242–448)	9.9 (7.7–12.3)	6 (4.8–7.3)	3.6 (3.6–3.6)					10 (6.8–13.2)	3.7 (3.6–3.7)	24.2 (21.2–27.2)	3.9 (3.8–3.9)
Cyclohexanone	183 (167–199)	6.4 (5.6–7.3)									9.7 (9.3–10.1)	0.3 (0.2–0,3)
Dimethyl disulphide	103 (82–125)	69 (57–82)	10 (8.7–11.3)	17.5 (16.8–18.1)	34.2 (32.8–35.6)	30.4 (29.6–31.1)	8.8 (7.5–10)	16.9 (16.2–17.5)	18.3 (16.2–20.4)	21.9 (20.8–23)	69.8 (66.3–73.3)	49.9 (48–51.9)
3-Hydroxy-2-Butanone	66.8 (43.7–89.9)	< 0.01 ppb							1.9 (1.5–2.2)	< 0.01 ppb	86.6 (76.6–96.6)	< 0.01 ppb
2-Methylfuran	26.2 (15.3–37.1)	< 0.01 ppb	3.9 (3.5–4.4)	< 0.01 ppb					3.4 (2.3–4.5)	< 0.01 ppb	13.8 (11.9–15.7)	< 0.01 ppb
2-Methylpentane	15.3 (10.4–20.2)	0.9 (0.1–1.8)	6 (5.2–6.8)	< 0.01 ppb					51.3 (29.2–73.4)	7.5 (3.4–12.1)	27.8 (24.1–31.5)	3.1 (2.5–3.8)
3-Pentanone	272 (207–338)	< 0.01 ppb									46.7 (42.7–50.7)	< 0.01 ppb

Results are shown as means (95% confidence interval) in millivolt [mV] and [ppb]. Total intensity of volatile organic compound was calculated on the basis of monomer and double dimer intensity

Participating patients had a mean age of 61 [± 16 SD] years, weight of 80.6 [± 16 SD] kg, and height of 172 [± 13 SD] cm. A total of 73 peaks were detected from patients, whereas individual measurements showed 44, 45, 55, 58, and 59 signals, respectively. 36 peaks were identified in the exhalome of all patients, whereas 14 signals were seen in but a single patient (Table 4).

**Table 4:** Main diagnosis, anaesthetics, and volatile organic compounds in the exhalome of five critical care patients

	Patient 1 59 VOCs	Patient 2 58 VOCs	Patient 3 55 VOCs	Patient 4 45 VOCs	Patient 5 44 VOCs
Diagnosis	Haemorrhagic shock, Peripartum atonic bleeding	Sepsis, Perforated sigmoid diverticulitis	Polytrauma, Brain injury	Sepsis, Mamma carcinoma	Femur fracture, Respiratory insufficiency
Sedation	Propofol and Remifentanil	Piritramide	Propofol and Remifentanil	Propofol and Remifentanil	Propofol and Remifentanil
VOCs in	P14, P30, P58, P62, P68	P13, P18, P67, P74	P69, P70, P71, P73		P61
1 patient					
(*n* = 14)					
VOCs in 2 patients (*n* = 9)			P54		
	P45, P57		P45, P57		
	P3, P6, P47, P59, P60, P63				
VOCs in 3 patients (*n* = 7)	P5		P5	P5	
		P11		P11	P11
		P38			
		P65, P66	P65, P66		P65, P66
	P51, P64				
VOCs in 4 patients (*n* = 7)	P24				
	P48		P48	P48	P48
	P4, P16	P4, P16	P4, P16		P4, P16
	P17, P35, P76				
VOCs in all patients (*n* = 36)	P1, P2, P7, P8, P9, P10, P12, P15, P19, P21, P22, P23, P25,				
	P26, P27, P28, P31, P32, P33, P34, P36, P37, P39, P40, P41,				
	P42, P43, P44, P46, P49, P50, P52, P53, P55, P56, P72				

36 compounds are detectable in all patients, respectively. Other volatiles are merely seen in 1, 2, 3, or 4 patients

Inspiratory gas supplied by an intensive care respirator yielded 12 distinct signals without distinction amongst the three tested ventilators. There were 4 peaks detected in oxygen and 5 in air from the hospital’s central gas supply at each tested distribution point. Oxygen from cylinders revealed 17 signals. Ambient air from the intensive care unit yielded 34 unique signals.

All detectable signals in oxygen from the central gas supply (dimethyl disulphide monomer, methanol, and two unknown compounds) were found in inhaled and exhaled gas as well as in compressed and room air. There were also 31 out of 34 signals from ambient air detectable in the exhalome of patients. 2-ethyl-1-hexanol and P75 were seen only in room air. The only volatile organic compound identified in inspired but not in expired gas (*resorbed* compound) was a monomer of isoprene. In contrast, isoprene dimer was merely seen in exhaled gas and in only one patient.

Eleven of the twelve inhaled volatiles were exhaled as well. Two unknown compounds (P67 and P74) were expired in similar concentrations to inspired gas and therefore designated as *unaffected* compounds; that is, they had expired-to-inspired peak intensities of 1.1 for P67 and 0.8 for P74. On the other hand, nine signals were seen at greater intensities in expired than in inspired gas and therefore termed *expired* compounds (acetone monomer, dimethyl disulphide monomer, methanol, 2-methylfuran, 2-methylpentan and four unknown compounds). In these 11 peaks, we recorded intensities between 3.1 (P72 and P74) and 45 mV (Methanol) for inhaled and peak intensities between 2.4 (P74) and 107 mV (Methanol) for exhaled gas. The derived *patient-to-inspired gas ratios* ranged from 0.8 (*unaffected* compounds) to 11.8 (*expired compounds*) and are summarised in Table 5. We observed 7 *contaminating* volatiles with *patient-to-inspired gas ratios* < 5.

**Table 5:** Volatile organic compounds detectable in inhaled and exhaled gas, derived patient-to-inspired gas ratios and corresponding classification into unaffected volatiles (patient-to-inspired gas ratio 0.5–1.5), and expired volatiles (patient-to-inspired gas ratio > 1.5)

IMS-Peak	Volatile Organic Compound	Patient-to-Inspired Gas Ratio	Classification	Retention Time	Drift Time
P74	*Unknown*	0.8	unaffected	41.5	0.697
P67	*Unknown*	1.1	unaffected	17.8	0.506
P51	*Unknown*	1.7	expired	38.7	0.497
P66	*Unknown*	1.9	expired	21.5	0.506
P32	Methanol	2.3	expired	0.0	0.478
P50	*Unknown*	2.5	expired	20.3	0.496
P36	2-Methylpentane	2.6	expired	9.1	0.510
P72	*Unknown*	5.0	expired	42.8	0.701
P35	2-Methylfurane	6.7	expired	4.8	0.540
P15	Dimethyl disulphide *monomer*	8.8	expired	8.0	0.498
P1	Acetone *monomer*	11.8	expired	3.0	0.498

The retention time (RT) and drift time (1/K0) describe the position of the peaks in the IMS-chromatogram. 7 volatile organic compounds yielded patient-to-inspired gas ratios <5 and were therefore designated as contaminants in expired air

**Fig. 2:**
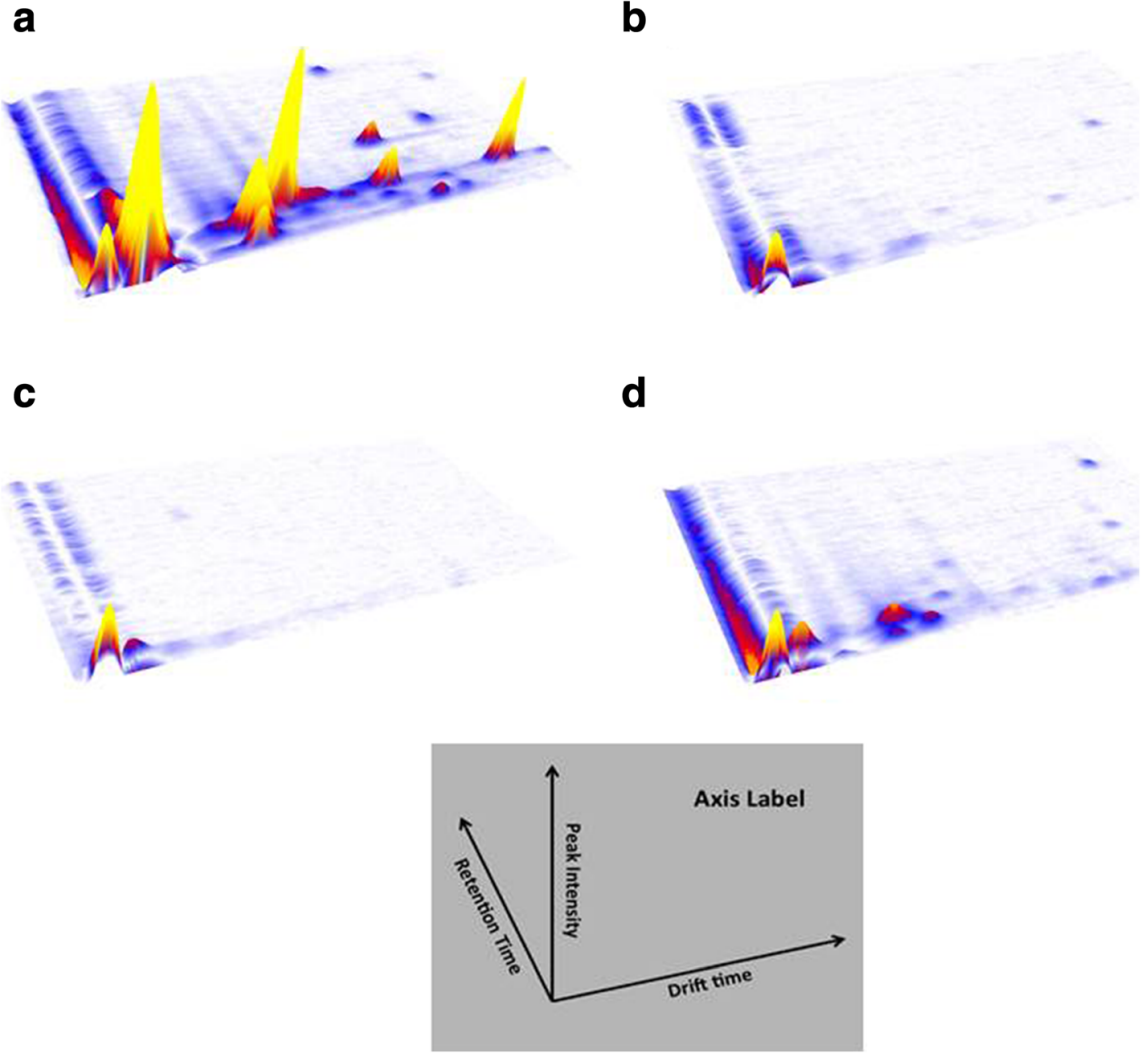
Three-dimensional ion-mobility spectrometry chromatograms for patients (a), inspired gas from a ventilator (b), oxygen from central gas supply (c) and ambient air of an intensive care unit (d). Volatile organic compounds are characterized according to *retention time, drift time, and peak intensity* in the chromatogram. Interestingly, most volatiles in inspired gas (b) exhibit similar small drift times and thus, are depicted merely at the edge in the IMS-chromatogram

**Fig. 3:**
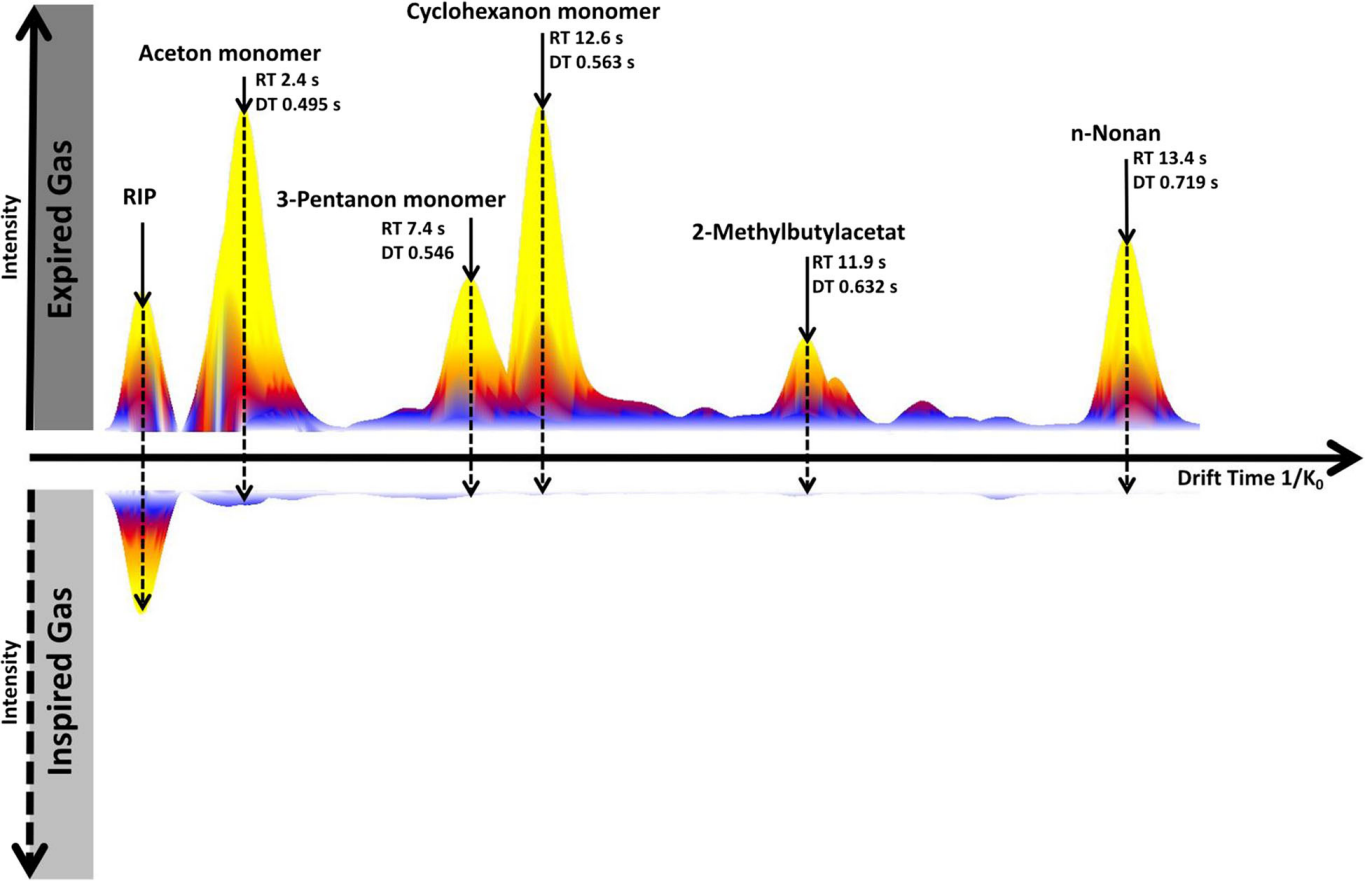
Direct and exemplary comparison of IMS chromatogram sections (two-dimensional view, different *retention times* are displayed in one plane) of inspired gas from respirator (directed downwards) and patient’s exhalome (directed upwards). Intensities of the selected exhaled compounds (peak heights) are considerably greater than inhaled concentrations. RIP = Reactant Ion Peak (Ionization of nitrogen and oxygen forms reactant ions and are always detectable, regardless of contamination)

There were a total of 71 *expired* signals (Table 2). Examples of three-dimensional ion-mobility spectrometry chromatograms are shown in Fig. 2 for the exhalome from patients, gas from ventilators, oxygen from the hospital’s central gas supply, and ambient air of the intensive care unit. Figure 3 compares inspired and expired gas in a typical patient.

## Discussion

There was significant variation in volatiles detected under various sampling conditions, but all gas sources were contaminated to some degree; specifically, we identified 17 signals from compressed oxygen cylinders, 12 signals from mechanical ventilators, 4 signals in oxygen from the central gas supply, and 5 signals in compressed air from the central gas supply. The central gas supply accounted for 4 signals found in ventilator gas. It can be presumed that volatile organic compounds in inhaled air originate from air being used for manufacturing oxygen and compressed air or derive from piping, seals, or respirator. Interestingly, nine of the 12 peaks detected in ventilator gases were detectable in room air as well — although no ambient air is supposed to be drawn into our ventilators. How these compounds got into ventilator gases remains unclear. Thirty-one of 34 detected signals in room air were also detectable in patients’ exhalome. Most likely, extubated patients, visitors, and staff exhale these compounds into the ambient atmosphere. However, it should be noted that we detected monomers and dimers in our sample collection. Thus, aforementioned unknown signals might include monomers and dimers, reducing the reported amount of unknown volatile organic compounds.

Analysis of the exhalome in ventilated critically ill patients shows promising approaches in detecting biomarkers, especially in the field of lung infections, to some extent ventilator associated and a common healthcare problem. Fowler et al. showed that volatiles in expired air of intubated and ventilated patients were able to classify breath profiles of patients with and without significant pathogen load in the lower respiratory tract [10]. Schnabel and colleagues detected 12 volatiles in ventilated critically ill patients that correctly discriminated between ventilator-associated pneumonia and the control group with high sensitivity and specificity [11]. Nevertheless, both authors did not evaluate background contamination sufficiently to prevent confounding by exogenous volatiles. Filipiak et al. demonstrated that appearance and concentration profile of pathogen-derived metabolites in expired air of ventilated patients with confirmed ventilator-associated pneumonia correlates with the presence of a particular pathogen [12]. They stated that several hours of continuous ventilation prevents bias of confounding exogenous contaminants like plastic-derived substances from tubing and ventilator by decreasing the concentration to levels prior to ventilation [12–14]. Risby et al. stated that continuous ventilation with pure gas mixture prior to breath sampling was an effective method for elimination of exogenous compounds from exhaled air [15]. In our opinion, both approaches exhibit methodological deficiencies: evaporation of volatiles from tubing and ventilator is impossible to anticipate. Furthermore, pure gas mixture from central gas supplies is not available as our results show. Gao et al. defined VOC profile being able to distinguish between lower respiratory tract infection, colonisation, and absence of acinetobacter baumannii pathogens in ventilated critically ill patients. However, they did not simultaneously detect inspired gas for background correction [16]. Regardless of the subject, normalisation of expired gas for inspired confounders is mandatory.

In expired air of ventilated critically ill patients, we reported several volatile organic compounds with notable intensities: acetone, 3-pentanone, cyclohexanone, and 3-hydroxy-2-butanone. Acetone monomer and dimer are among the main components of exhaled breath. Acetone dimer was also detected in ambient air which is consistent with Bessonneau and colleagues who report that air in hospitals contains more acetone than other public buildings or private homes [17]. Nevertheless, it should be noted that expired acetone might decrease during critical illness, as we demonstrated in a previous study [5]. For this reason, our findings are specific for critically ill patients due to inflammation and sepsis. Additionally, we detected expired 3-pentanone in high intensities. We also found monomer and dimer forms at lower intensities in room air, possibly the result of exhalation by staff and visitors. Considering dimerization of several volatiles and thus twofold number of molecules in dimer clusters, acetone is the most abundant volatile organic compound in expired gas. Cyclohexanone had one of the highest intensities in expired gas but was detectable in room air in lower intensities. This volatile compound is widely used as an adhesive solvent during manufacture of medical devices which may explain its presence in ambient air [18]. Still, it remains uncertain why cyclohexanone was not present in inspiratory gas that is passing through the ventilator circuit. It is possible that evaporation might depend on running time of ventilator and tubing system and that washout kinetics cause a decline in concentration. Kischkel et al. detected cyclohexanone in medical synthetic air and much more in expired air under mechanical ventilation that had passed through an endotracheal tube, but not in ambient air [14]. They stated that cyclohexanone originates from the material of the endotracheal tube, supporting our findings with high intensities in expired air but not in inspiratory gases. 3-hydroxy-2-butanone, also known as acetoin, revealed substantial intensities in expired air. *Staphylococcus aureus*, a common pulmonary pathogen in ventilated patients, is well known to produce a characteristic profile of 3-hydroxy-2-butanone and might be partly responsible for the evidence in exhaled breath [19, 20]. Furthermore, acetoin in expired air might be released as a result of cellular damage due to the reactive oxygen species [21]. However, we detected acetoin in room air in high intensities as well. 3-hydroxy-2-butanone is a known flavouring chemical, widely used for food, cigarettes, cosmetics, or detergents and detectable in the breath of healthy individuals as well, which might explain our findings [21]. Our results are generally consistent with Filipiak et al. who determined that expired gas composition is altered by exogenous exposure including smoking and exposure to air pollutants. They identified 86 organic compounds in expired gas to tobacco smoke, most unsaturated hydrocarbons. Exposure to indoor-air contaminations and diet were identified as further contributing factors [22].

Inspired gas in our institution is polluted by six volatiles, all detectable in exhaled air as well: acetone, dimethyl disulphide, isoprene, methanol, 2-methylfuran, and 2-methylpentane. Sturney and colleagues detected acetone in the inspiratory limb of the respirator of intubated and ventilated patients in the intensive care unit as well [23]. They stated a correlation between inspired and expired acetone concentrations, possibly related to contamination of inspiratory samples by exhaled acetone in the inspiratory part of the ventilator in a rebreathing system. Otherwise, components of the respirator and breathing circuit itself might be the source of acetone [23]. These findings would support our detection of acetone in inspired air, but the exact origin remains unknown. In expired air, acetone is the main volatile in human breath and is produced endogenously by hepatic decarboxylation, mainly during lipolysis [23]. The occurrence is related to fasting, diet, patients with diabetes mellitus, and well described in critically ill patients [6, 23], explaining the high intensities of monomer and dimer we present.

We determined dimethyl disulphide in inspired air, gas supplies, and room air. This volatile sulphur compound is released by muscle cells in rats [24] and likewise by cultures of pseudomonas aeruginosa from patients with cystic fibrosis [25]. Nonetheless, we can only speculate about the detection of dimethyl disulphide in inhaled air.

Isoprene is also one of the most abundant volatiles in human breath and a byproduct of cholesterol biosynthesis [26]. Isoprene might be related to oxidative damage to the fluid lining of the lung and the body [27, 28]. Patients with pulmonary fibrosis showed significant higher peak intensities of isoprene compared to healthy subjects [21]. Interestingly, the concentration of isoprene in expired human air is age dependent and shows a circadian rhythm with a maximum in the morning and lower concentrations in the evening [26]. Schubert and colleagues detected isoprene in inspired air of mechanically ventilated patients as well, confirming our findings [29]. Yet, it should be mentioned that we detected isoprene in exhaled breath solely in a single patient, and that in low intensity. These findings are hypothetic and very unlikely. However, in using ion-mobility spectrometry for breath analysis, intensities of different volatiles are substance specific in itself. It is know that isoprene shows a weak signal at the detector of the IMS leading to a poor response even in higher concentrations of isoprene in humid exhaled breath. Protonated isoprene does not form hydrates or cluster like other volatiles and therefore can pass through the drift tube more rapidly. Moreover, the ion lifetime is short, leading to weak ion detection. Finally, presence of interfering ions might be another reason for poor detection of isoprene using ion-mobility spectrometry [30]. Furthermore, these findings have been described only for a few volatile organic compounds. Therefore, statements concerning isoprene intensities in the exhalome must be treated with caution.

Methanol is frequently used as a solvent or detergent. This volatile has been described as a typical outdoor air volatile and might therefore contribute to the presence in gas supplies and inspired air from the ventilator [31]. Moreover, methanol might originate from piping or seals of the ventilator circuit as well. Notably, methanol intensities in exhaled air were twice as high as detected in inspired air. This compound is a major endogenous breath metabolite and also present in expired air of healthy people [32]. A fraction of exhaled methanol was shown to be inhaled from the ambient atmosphere [33]. Elevated levels of this compound has been associated with liver cirrhosis in humans, interestingly decreasing after transplantation [34]. However, concerning volatile isoprene, methanol only leads to a weak response at the IMS detector, as stated previously. Methanol reacts with small hydrated hydronium ions but fails to further react with large ones due to their dipole moments [30]. Thus, quantitative statements comparing methanol intensities with other volatiles are challenging.

We determined 2-methylfuran in ventilator gas, room air, and expired air, but not in the central gas supply. This volatile is an odour component in cigarette smoke and is present especially in the exhalome of smoking subjects [35]. It is observed in ambient air and detectable in rainwater in considerable amounts [36]. The presence in inhaled air but not in the central gas supply suggests the release from components of the respirator.

In a previous animal study, we assigned 2-methylpentane, a branched-chain alkane and structural isomer of hexane, to be a ‘respirator peak’. We now report low intensities in ventilator gas as well. However, this volatile was not detectable in oxygen and compressed air from central gas supply as stated for 2-methylfuran. Therefore, 2-methylpentane might also be released by the ventilator. Yoshida demonstrated the pulmonary absorption of 2-methylpentane by inhalation in a rat model based on the knowledge of well-known diffusion [37]. To what extent inhaled 2-methylpentane affects concentrations in expired air also remains unknown. Filipiak and colleagues observed higher concentrations of 2-methylpentane in the breath of lung cancer patients compared to healthy controls [38], confirming our observation of 2-methylpentane in expired air in human subjects.

Propofol, an intravenous anaesthetic and frequently used for sedation in critical ill patients, can be detected in patients’ exhalome using IMS [39]. We detected propofol in all four patients who were given this intravenous anaesthetic for sedation.

We divided volatile organic compounds into three different groups: *expired* volatiles which originate from metabolism, *resorbed* compounds which originate outside the body and are absorbed, and *unaffected* compounds which are inert and thus present in comparable concentrations in inhaled and expired gas. We previously recorded 22 expired, 12 unaffected, and 3 resorbed volatiles in rats [6]. However, we now report 71 expired compounds, most likely because humans have more environmental influences, illnesses, metabolic differences, medications, and habits than rats. Twelve of the 22 expired substances in rats were also expired in humans.

Fortunately, most compounds detected in inspired gas were also found in expired gas from patients. Most are probably inert compounds that are unaffected by metabolism and thus unimportant for breath analysis. We also observed compounds that had considerably lower concentrations in expired than inspired gas, suggesting that they were resorbed and thus probably irrelevant for breath analysis. However, there were several signals with sustained higher concentrations in expired than inspired gas. These expired volatiles are presumably endogenously derived and thus potentially reflect the patient’s metabolic state. Overall, we identified 11 volatiles that could cause uncertainties in interpretation of the patient’s exhalome: acetone monomer, dimethyl disulphide monomer, methanol, 2-methylfurane, 2-methylpentane, and an additional 6 unknown compounds. However, only 7 compounds showed *patient-to-inspired gas ratios* < 5 and were therefore considered clinically important for contamination.

The obvious conclusion from our results is that expired concentrations of volatile organic compounds should be normalized for multiplexed inspired concentrations which are technically easy to assess. This approach is consistent with the recommendation of Philips and colleagues who proposed subtracting inhaled concentrations of volatile compounds from their expired concentrations [40], an approach that appears valid at the relatively low concentrations we observed [29]. We note, though, that the effects of inspired substance concentrations on expired concentrations depends on the blood-to-alveolar gradient which is not necessarily linear and can be influenced by shunt perfusion and dead space ventilation, especially in mechanically ventilated patients [41].

Spanel et al. proposed that all exogenous compounds are partially retained in the exhaled breath according to a close linear relationship between exhaled and inhaled concentrations. They defined *retention coefficients (α)*, with values between 0.1 and 1.0, specifically for each compound. When the *retention coefficient* is close to 1, such as for hydrocarbons and alkanes, inspired concentrations can simply be subtracted from exhaled concentrations by full amount as proposed by Phillips [40]. On the other hand, when the *retention coefficient* is close to 0.1, such as for water-soluble compounds, inspired concentrations can essentially be ignored in breath analysis [42]. Spanel and colleagues recorded *retention coefficients* for seven volatiles, ranging from 0.76 for pentane to 0.09 for deuterated water.

In some ways, our study has several limitations. First, ion-mobility spectrometry uses intensities (millivolt) as a unit of quantity rather than of concentrations. Therefore, comparing our results with other data might be difficult. Quantitative statements are partially speculative and not comparable to other studies because detector response of ion mobility spectrometer is substance specific. In any case, the existing recommendation line is unaffected by this: analysis of the exhalome in critical care patients requires normalisation of the expired to inspired signals. In addition, the data we present are specific for our intensive care unit (the ventilators we tested, our own hospital’s central gas supply) and probably not applicable in different settings. Secondly, as mentioned above, quantification and even correct detection of isoprene and methanol is impossible at high levels observed in expired air using ion-mobility spectrometry. Therefore, comparing intensities of these compounds to other volatiles is impossible and statements must be treated with great caution. Only abundances of the same particular compound can be compared between samples. Thirdly, we assume that volatile organic compounds will differ in other contexts, but our goal was not to exactly characterise the patterns in ambient air, gas cylinders, hospital central gas supply, or specific ventilators. Instead, it was to demonstrate that volatile organic compounds are ubiquitous and that any clinical measurement system will need to incorporate multiplexed measurements and compensate for inspired compounds.

While not all volatile compounds were detectable in every patient, nearly half were. In contrast, 14 volatile compounds were detected from single patients and nine others from just two patients. The extent to which the common or unusual compounds reflect normal or abnormal biology — or perhaps drug metabolites — remains largely unknown at this point. Much larger studies will be required to characterise patient-to-patient variability, not to mention how various diseases moderate the exhalome, neither of which was a goal of the current study. In addition, further studies have to focus on the relationship between volatiles’ peak intensity in chromatogram and their normally used units in gases.

## Conclusions

Ambient air in critical care units as well as gas from compressed cylinders and from central hospital supplies are all contaminated with various volatile organic compounds. Consequently, gases from mechanical ventilators are as well. Future studies of the exhalome in mechanically ventilated patients should consider and compensate for background contamination.
